# Pulmonary Vein Stenosis Following Ablation for Atrial Fibrillation

**DOI:** 10.7759/cureus.110152

**Published:** 2026-06-02

**Authors:** Gráinne O'Brien, Adel Tachouli, Eirini Kasfiki

**Affiliations:** 1 Acute Medicine, Hull University Teaching Hospitals NHS Trust, Hull, GBR

**Keywords:** atrial fibrillation, catheter ablation, dyspnoea, haemoptysis, pulmonary vein stenosis

## Abstract

Pulmonary vein stenosis (PVS) is a rare but potentially serious complication of catheter ablation for atrial fibrillation (AF), often presenting with non-specific respiratory symptoms and initially misdiagnosed. Early recognition is crucial to reduce the risks of progressive pulmonary hypertension, infarction, and long-term morbidity. We report a 65-year-old woman who presented with progressive dyspnoea on exertion, marked reduced exercise tolerance, lethargy, chest pain , and new onset haemoptysis. These symptoms began five months following an ablation procedure for symptomatic AF. Initial evaluations by primary care and cardiology teams attributed symptoms to lower respiratory tract infection or drug toxicity. On admission to the hospital, vital signs were normal, and routine investigations were unremarkable. Cardiac CT with pulmonary venography confirmed high-grade ostial stenosis (> 90%) of both left pulmonary veins. The patient underwent elective percutaneous pulmonary venoplasty with stenting under general anaesthesia. Post-procedural follow-up showed marked improvement in symptoms and patency of the left pulmonary veins on imaging. PVS secondary to catheter ablation is a rare but important post-ablation complication, which is frequently misdiagnosed due to non-specific symptoms. High clinical suspicion, early imaging, and timely endovascular intervention are important to optimise outcomes and minimise restenosis risk. Clinicians should consider PVS in any patient presenting with progressive dyspnoea, haemoptysis , or chest pain in the months following atrial ablation. This case highlights the importance of awareness to enable early diagnosis and treatment, preventing potential morbidity.

## Introduction

Atrial fibrillation (AF) is the most common sustained cardiac arrhythmia worldwide and is associated with significant morbidity, including stroke, heart failure, and reduced quality of life [1]. Catheter-based ablation is an established rhythm-control strategy for patients with symptomatic AF who remain refractory to medical therapy [1]. The procedure aims to electrically isolate the pulmonary veins, which are recognised as the major source of ectopic electrical activity that initiates and maintains AF [1]. Pulmonary veins normally return oxygenated blood from the lungs to the left atrium, and preservation of unobstructed pulmonary venous flow is essential for maintaining normal pulmonary circulation and gas exchange. Although generally safe and effective, the ablation procedure carries recognised complications, one of which is pulmonary vein stenosis (PVS) [2].

PVS is an uncommon but potentially serious complication of AF ablation, reported in 0.29-3.4% of cases [2,3]. It is thought to result from thermal injury to the pulmonary venous endothelium during ablation, leading to inflammation, fibrosis, and progressive luminal narrowing [3]. Progressive pulmonary venous obstruction can impair drainage from affected lung segments, causing pulmonary venous hypertension, oedema, and infarction, which may become irreversible if severe and left untreated [2,3]. Clinical presentation is often delayed and non-specific, with symptoms such as dyspnoea, cough, chest discomfort, or haemoptysis, which may mimic more common respiratory conditions and lead to delayed diagnosis [2].

We present a case of severe left-sided PVS presenting with progressive respiratory symptoms several months after catheter ablation for AF, highlighting the diagnostic challenges associated with this rare complication and the importance of maintaining a high index of suspicion for timely diagnosis and intervention.

## Case presentation

A 65-year-old female presented to the emergency medical services with progressive dyspnea on exertion for a few weeks, marked reduced exercise tolerance, reported lethargy for the same duration, and new-onset haemoptysis for the last three days.

She was an ex-smoker of 12 pack-years with no other significant respiratory history. She worked as a front counter officer (support staff) for a regional police department, with no history of exposure to chemicals, dust, or asbestos. She had no history of respiratory problems prior to this presentation. She had a pet dog for many years with no animal allergies and no travel history of note. There was no family history of allergies or malignancy. There were no identified risk factors for pulmonary embolism.

Her past medical history included gastro-oesophageal reflux disease and AF. Surgical history included breast augmentation 20 years prior to this presentation and pulmonary vein isolation with radiofrequency ablation for AF five months prior. This procedure was straightforward, with no complications, including no issues with the left pulmonary veins. At the time, the pulmonary venous anatomy was reported as normal with all four pulmonary veins electrically active at baseline.

Her regular medications included bisoprolol 1.25 mg BD, edoxaban 60 mg OD, atorvastatin 20 mg OD, esomeprazole 20 mg OD, and linaclotide 290 mcg OD.

A detailed history of clinical events was sought, and a timeline was documented in the notes describing sudden onset, rapidly progressing dyspnoea on exertion.

She originally presented to primary care with reported shortness of breath accompanied by dry cough and fatigue for three weeks' duration. On assessment, the patient's oxygen saturations were 97% on room air, and clinical examination revealed no red flags for pulmonary embolism. At the time, she was taking amiodarone, as well as the above-mentioned medications. Her primary physician treated the episode as a lower respiratory chest infection with a course of oral antibiotics with a view to testing B-type natriuretic peptide (BNP) if symptoms persisted.

**Figure 1:**
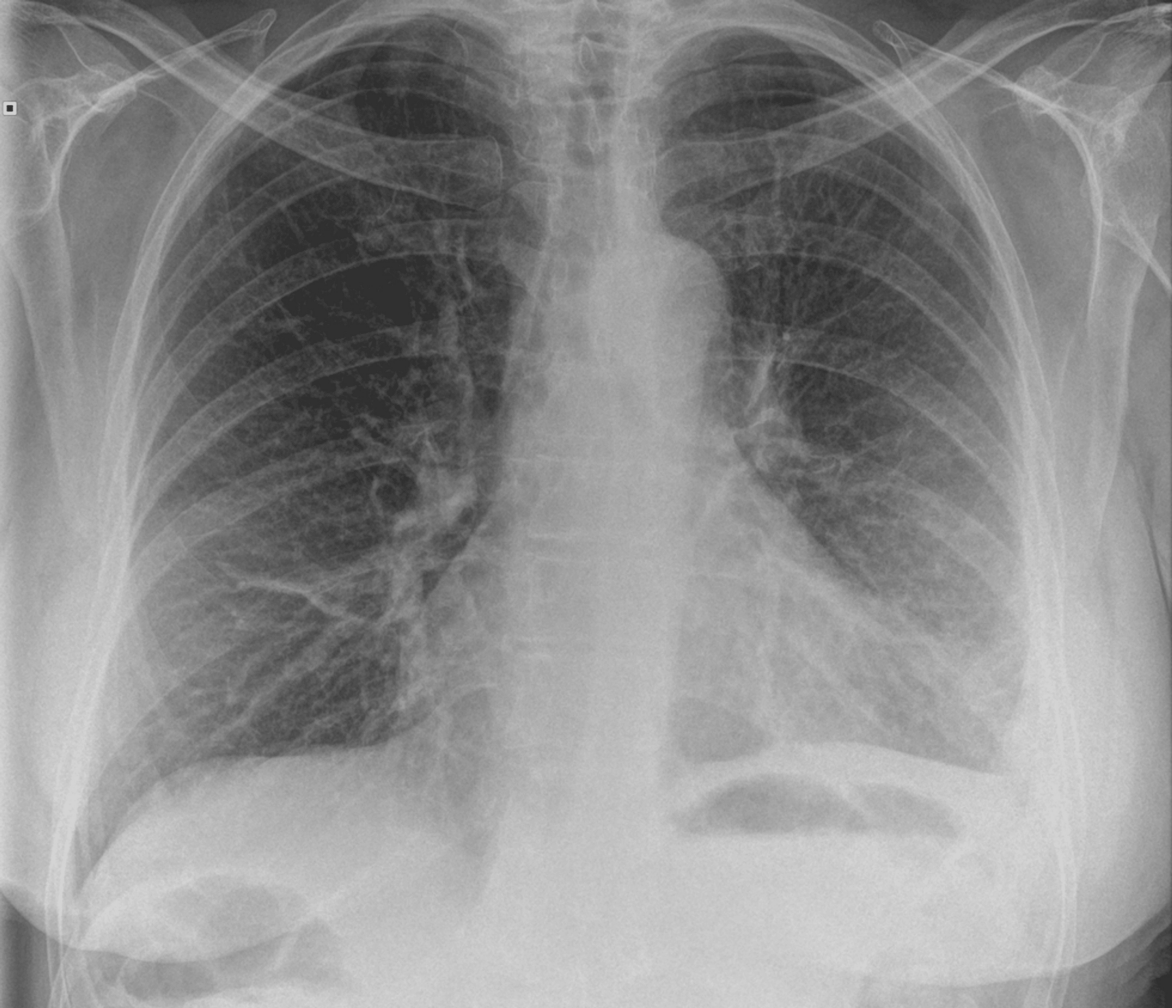
Chest radiograph demonstrating left-sided basal consolidation.

She presented to primary care services two weeks later with worsening dyspnoea and reduced exercise tolerance. Again, respiratory examination was unremarkable, and oxygen saturations were 97% on room air with no clinical suspicion of pulmonary embolism. Routine blood tests carried out were normal, including a BNP of 141 pg/mL (reference range of 0-400 pg/mL), and chest X-ray showed some consolidation at the left base (Figure 1). The symptoms and signs were attributed to the recently treated lower respiratory tract infection, and no further action was deemed necessary. The patient was discharged.   

Her dyspnoea continued to progress, and meanwhile, the cardiology team reviewed as routine follow-up post pulmonary vein ablation for her AF. A 12-lead electrocardiogram showed a normal sinus rhythm. Her symptoms were attributed to possible drug-related pulmonary toxicity. Amiodarone was discontinued in case of drug-related pulmonary toxicity, and the patient was discharged from the clinic. No lung diffusion testing was undertaken at this time.

Two weeks later, the patient presented to emergency medical services with progressive, exertional dyspnoea (as described above), marked reduced exercise tolerance (describing a drop from 1 km on the flat to less than 200 m in the last five weeks), pleuritic left-sided chest pain present intermittently throughout the period of the worsening dyspnoea, and new-onset haemoptysis of a few days' duration.

A comprehensive history was sought and revealed the above sequence of events; sudden onset of dyspnoea five months post-pulmonary vein isolation, with rapid progression of symptoms in the last month. On systematic enquiry, she denied dizziness, weight loss, night sweats, fever, or other infective symptoms; she had no orthopnoea or paroxysmal nocturnal dyspnoea.

Her vital signs were normal, with a blood pressure of 118/63 mmHg, heart rate of 77 bpm, regular and good volume pulse with normal character, respiratory rate of 17 bpm at rest, temperature of 37.1 °C, and oxygen saturations of 96% on room air at rest.

Clinical examination revealed chest wall tenderness over the left apex, axilla, and shoulder blade, reduced air entry on auscultation of the left base, and dullness to percussion over the same area. Cardiovascular examination was normal; there were no signs of pulmonary hypertension or heart failure.

**Figure 2:**
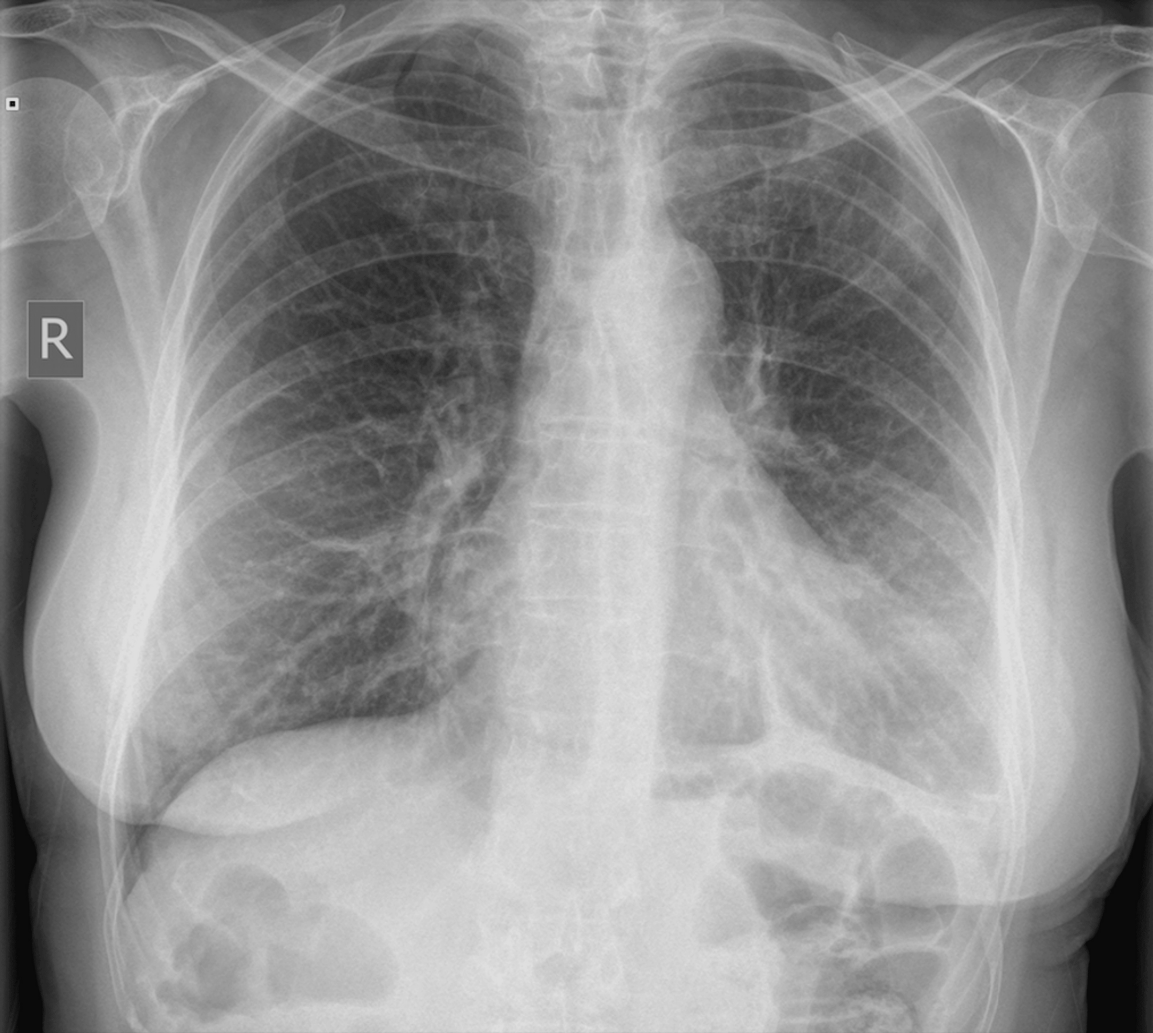
Chest X-ray demonstrating patchy left basal opacification with additional infiltrates in the left upper zone; right lung field clear and cardiomediastinal contours within normal limits.

Investigations revealed an elevated CRP, raised D-dimer, and mild hypoalbuminaemia, with other blood tests, including white cell count within normal limits, making infection a less likely differential at this stage (Table 1). Chest X-ray showed patchy left basal opacification, with additional infiltrates in the left upper zone, the right lung field clear, and cardiomediastinal contours within normal limits (Figure 2).

**Table 1:** Laboratory investigation results on admission to hospital showing raised CRP, D-dimer, and mild hypoalbuminaemia.

Test	Result	Reference range	Units
C-reactive Protein (CRP)	46	0-8	mg/L
D-dimer	999	0-500	ng/mL
Albumin	31	36-48	g/L
White cell count (WCC)	6.3	4-11	×10⁹/L

**Figure 3:**
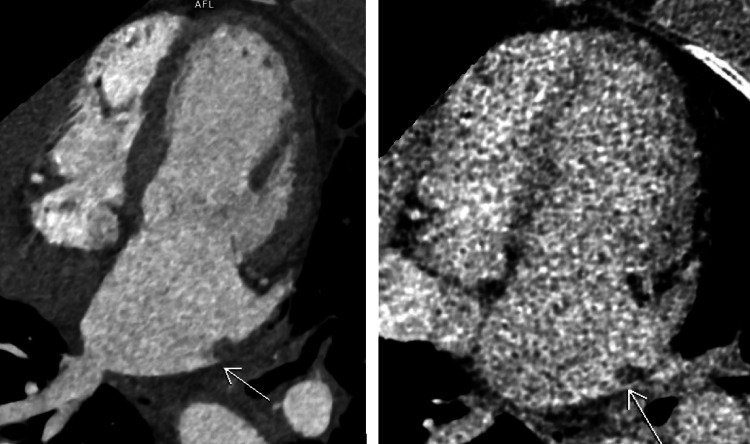
Superior left pulmonary vein, arterial phase showing a narrowed ostium (left arrow) and delayed phase showing a thin sliver of contrast in the distal lumen (right arrow).

**Figure 4:**
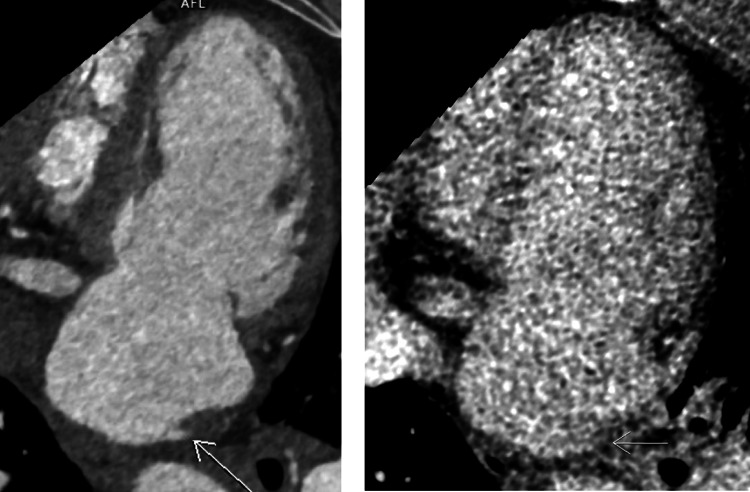
Inferior left pulmonary vein arterial phase showing a narrowed ostium (left arrow) and delayed phase showing no appreciable contrast in the distal lumen (right arrow).

To investigate further, a combined CT pulmonary angiogram and CT coronary angiography (CTCA) was performed, ruling out acute thromboembolism. However, specialised post-processing reconstruction of the CTCA dataset was performed. This utilised an ECG-gated, small-field-view protocol for the coronary arterial and delayed phases. The images showed a visually evident severe narrowing of the superior and inferior left pulmonary vein ostia. These ostia appeared very narrow and slit-like on the coronary arterial phase. On the delayed phase, there was a sliver of contrast opacification in the distal superior vein, indicating severe narrowing, but there was no opacification of the inferior left pulmonary vein distally, indicating a very high-grade stenosis (near total) > 90% (Figures 3-4).

A diagnosis of severe left-sided pulmonary vein stenosis (PVS) secondary to prior AF ablation was established.

Management options presented to the patient included surgical repair versus endovascular intervention. After multidisciplinary discussion, intervention was recommended due to symptomatic, progressive disease and high-grade, superior, and inferior left-sided PVS. The patient opted for treatment with elective percutaneous pulmonary venoplasty, with stenting under uneventful general anaesthesia.

Clinical follow-up with CT imaging at three months post intervention showed a marked improvement in dyspnoea and functional capacity. CT thorax showed sustained patency of the left pulmonary veins and resolution of the pleural effusion.

## Discussion

PVS is a recognised complication of catheter ablation for AF ​[2]. The incidence of symptomatic vein stenosis post isolation varies between 0.29% and 3.4% ​[3]. Stenosis is thought to develop due to thermal injury of the endothelium from the ablation, leading to inflammation, collagen deposition, neointimal hyperplasia, and progressive fibrosis, causing vein narrowing ​[3]. Left-sided pulmonary veins are more likely to be affected than right-sided veins [3], as this case presented.

Catheter ablation using thermal energy sources, including radiofrequency ablation and cryoballoon ablation, achieves pulmonary vein isolation through tissue heating or freezing, respectively. However, the indiscriminate nature of thermal injury may damage adjacent atrial and extracardiac structures, resulting in complications such as PVS, phrenic nerve injury, and atrio-oesophageal fistula [4,5]. In contrast to catheter ablation, pulsed-field ablation (PFA) is a largely non-thermal modality that induces myocardial cell death via irreversible electroporation while sparing surrounding tissues, including the pulmonary veins. Early studies have demonstrated favourable safety outcomes with minimal clinically significant pulmonary vein narrowing following PFA [4,5].

Clinical symptoms develop months following ablation, reflecting the progressive fibrotic process ​[6]. Our patient presented with all the described symptoms of a PVS diagnosis: exertional dyspnoea, cough, chest pain, and haemoptysis [7].

The patient was initially misdiagnosed by both primary care and the cardiology teams; however, this is not uncommon. Approximately 33% of patients were initially misdiagnosed with conditions such as bronchitis, pneumonia, or malignancy ​[3,6]. This is attributed to the fact that symptoms are non-specific and often point towards underlying respiratory pathology ​[2].

Treatment of PVS includes primarily endovascular intervention under direct radiological guidance, which consists of balloon angioplasty and stent implantation, with stenting achieving higher success rates than balloon angioplasty alone. Post endovascular repair, patients require follow-up imaging, typically with CT, to monitor for restenosis ​[2], which is common following initial intervention.

## Conclusions

PVS remains a recognised complication of catheter ablation for AF. Although advances in ablation technology and technique have reduced its occurrence, it should remain within the differential diagnoses in any patient presenting with progressive dyspnoea in the months following pulmonary vein isolation. Maintaining a high index of suspicion is crucial, as early recognition and timely intervention are key to optimising outcomes and reducing the risk of restenosis. Ultimately, awareness of this complication ensures that opportunities for early diagnosis and treatment are not missed, reducing disability and procedural complications post-endovascular repair.

This case highlights the diagnostic challenge posed by PVS, a rare but recognised complication of catheter ablation for AF. The patient presented with the characteristic symptoms of PVS within the expected time frame following the procedure; however, the non-specific nature of these symptoms can mimic more common respiratory conditions and contribute to delayed diagnosis. Following endovascular repair, the patient demonstrated good clinical and radiological recovery with minimal residual symptoms on follow-up. This case emphasises the importance of maintaining a high index of suspicion for PVS in patients presenting with unexplained respiratory symptoms weeks to months after pulmonary vein isolation, as early recognition and intervention may improve outcomes and reduce the risk of progression or restenosis.
